# Olfactory receptor OR51B5 suppressed esophageal cancer progression through activates Calcium / N-Ras signaling

**DOI:** 10.1038/s41419-025-07769-9

**Published:** 2025-06-16

**Authors:** Fan Yang, Jiaqi Yang, Chengbo Zhu, Tianyi Ding, Xiaoyu Zhang, He Zhang

**Affiliations:** 1 State Key Laboratory of Cardiology and Medical Innovation Center, Province Key Laboratory of Organ Development and Epigenetics, Institute for Regenerative Medicine, Shanghai East Hospital, Frontier Science Research Center for Stem Cells, Jinggangshan Enclave Laboratory, School of Life Science and Technology, Tongji University, Shanghai, P. R. China; 2 Clinical Medical Research Center, Affiliated Hospital of Jinggangshan University, Medical Department of Jinggangshan University, Ji’an, P.R. China; 3 School of Life Science, Jinggangshan University, Ji’an, P.R. China

**Keywords:** Epigenetics, Cancer

## Abstract

The transcriptional regulation of olfactory receptors (ORs) plays a critical role in various biological processes, and has recently been considered a potential therapeutic target for cancer treatment. Esophageal cancer (EC) is a highly invasive neoplasm with dismal prognosis, but the specific roles of ORs in EC remain largely unexplored. Here, we developed a comprehensive workflow to identify potential functional olfactory receptor family 51 subfamily B member 5 (*OR51B5*) and demonstrated that *OR51B5* locus acted as a key spatial element contributing to the progression of esophageal cancer. Moreover, we showed that the CTCF-EZH2 enhanced the trimethylation of lysine 27 of histone H3 (H3K27me3) and increased repressive and closed chromatin state at the *OR51B5* promoter region. Subsequently we demonstrated that closed chromatin impaired the entry of RNA polymerase II and inhibited the transcription of *OR51B5*, thereby causing N-Ras activation and promoting tumor cell proliferation and metastasis. Our study provides an alternative workflow for discovering critical regulatory sites for control tumorigenesis, and reveals a novel OR51B5 triggering mechanism underlying esophageal cancer progression.

## Introduction

Olfactory receptors proteins are important members of the G protein-coupled receptor (GPCR) family, which are widely expressed in various tissues and cells, and their abnormal expression or abnormal function may be closely related to the occurrence and development of neurodegenerative diseases and metabolic diseases [1–3]. Studies have shown that ORs play an important role in physiological processes, including sperm chemotaxis, muscle differentiation, and angiogenesis [4–6]. Activation of Olfr78 by short-chain fatty acids (SCFAs) triggers cAMP signaling, inducing renin gene transcription and consequently modulating systemic blood pressure [7]. So far, the role of olfactory receptor proteins has received increasing attention in the physiological and pathological processes of human diseases and dysplasia.

Olfactory receptors contribute to tumorigenesis by modulating cell proliferation, apoptosis, migration, and metabolism [8, 9]. Certain OR family members function as oncogenes or tumor suppressors across various malignancies, playing pivotal roles in carcinogenic signaling networks [10]. In liver cancer cells, engagement of OR1A2 by citronellal diminishes calcium signaling and suppresses cell proliferation [11]. Similarly, heightened expression of OR2J3 following exposure to sunlight in non-small cell lung cancer triggers apoptosis and inhibits cell proliferation [12]. Esophageal cancer is the eighth most commonly diagnosed cancer and the sixth leading cause of cancer-related mortality worldwide, characterized by late-stage diagnosis and poor prognosis [13, 14]. However, the role of olfactory receptors underlying esophageal carcinogenesis remains to be further investigation.

Spatial chromatin dynamics lead to activation and repression of their target genes [15]. Once chromatin was turned on, transcription factors and other regulatory proteins could access open chromatin regions, thereby promoting transcription. Conversely, closed chromatin accumulates repressive status, leading to obscured chromatin-binding sites and transcriptional silencing [16, 17]. Similar epigenetic mechanisms govern the intricate transcriptional regulation of olfactory receptor genes, operating across multiple layers from one-dimensional to three-dimensional levels, thereby illustrating the broad influence of spatial chromatin architecture on gene transcription [18]. Linking mRNA to Chromatin Architecture (LiMCA) indicates that cis-enhancers can activate the expression of olfactory receptor genes [19]. Furthermore, MeCP2 regulates the transcription of *OR* and *TAS2R* genes through interaction with H3K9me3, which may be implicated in the development of sporadic Alzheimer disease [20].However, it remains uncertain whether chromatin dynamic changes can regulate transcription of olfactory receptor genes, especially in esophageal tumorigenesis.

Most importantly, identifying efficient chromatin sites within chromatin dynamics that may be associated with esophageal cancer progression remains an open question. As of today, various strategy has been developed to resolve this intriguing problem. Utilizing Hi-C and RNA-seq employed simultaneously (HiRES) strategy, extensive chromatin sites in chromatin dynamics are found to be reconfigured before transcriptional activation. The SimpleDiff further identified 98 genes where expression changes correlate with dynamic chromatin rearrangements [21]. In addition, transposase-mediated chromatin accessibility sequencing (ATAC-seq) strategy reveals 11,762 differential accessible peaks between invasive lobular carcinoma (ILC) and invasive ductal carcinoma (IDC) in primary breast cancer. *EGR1*, *SOX* and *TEAD* families are significantly influenced by ILC-associated changes of open chromatin [22]. Single-cell RNA-seq strategy demonstrates that knockout of *METTL3*, *TET1* and *FXR1* genes alters chromatin accessibility in specific regions, reducing oncogenes *FAT4* and *SAMD9L* expression, thereby promoting esophageal cancer cell proliferation [23]. However, plenty of functional dynamic chromatin locus potentially involved in this process remain undiscovered, indicating the alternative strategy for identifying relevant chromatin sites need to be further developed.

In this study, we successfully established a comprehensive workflow (CiFR) to identify the *OR51B5* locus served as a critical chromatin site contributing to esophageal cancer progression, and revealed that OR51B5 is a novel esophageal cancer suppressor. We observed that *OR51B5* overexpression enhances intracellular Ca²^+^ signaling, reduces N-Ras expression, and inhibited the growth and metastasis of esophageal cancer.

## Results

### Closed chromatin of *OR51B5* site was found at the chr11p15.4 locus in ESCC

**Fig. 1:**
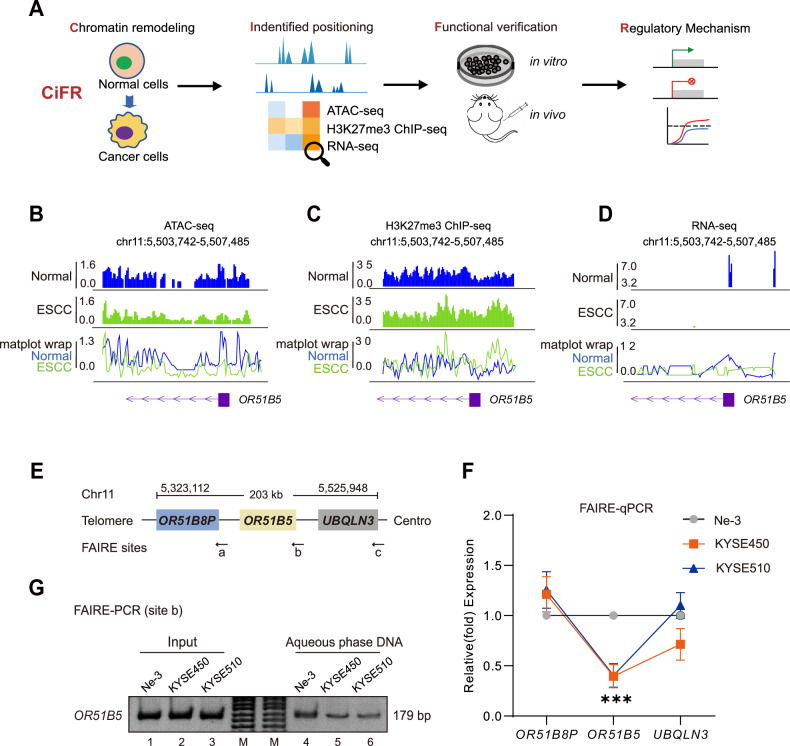
Closed chromatin of *OR51B5* site was found at the chr11p15.4 locus in ESCC. **A** Diagram of CiFR workflow. **CiFR**: **C**hromatin remodeling, **i**dentified positioning, **F**unctional verification, **R**egulatory mechanism. **B** Chromatin dynamics status at the chr11p15.4 (chr11:5,503,742–5,507,485) locus with ATAC-seq (GSE162380). **C** Chromatin dynamics status at the H3K27me3 ChIP-seq at the chr11:5,503,742–5,507,485 (SRP312409). **D** Expression status at the RNA-seq at the chr11:5,503,742–5,507,485 (GSE194116). **E** Schematic diagram of the FAIRE sites at Chr11:5,323,112–5,525,948. Site a-c: three FAIRE sites of the *OR51B8P* promoter, *OR51B5* promoter and *UBQLN3* promoter. **F** Quantification of the FAIRE at Chr11: 5,323,112–5,525,948 of ESCC cells (KYSE450 and KYSE510) and human esophageal epithelial cell (Ne-3). All of the data are presented as the mean ± SD. *P* values were calculated by two-tailed Student’s *t* test. ***P < 0.001. **G** The FAIRE-PCR assay at *OR51B5* promoter (site b) of KYSE450 and KYSE510 cells and Ne-3 cell. Input DNA was used as a positive control.

To investigate efficient chromatin sites triggering tumorigenesis, we proposed a comprehensive workflow (CiFR) by integrating multiple analysis (Fig. 1A). Firstly, in cells with **C**hromatin remodeling, we employed H3K27me3 ChIP-seq, ATAC-seq and RNA-seq to identify potential targets with altered chromatin status at their promoter region (**i**dentified positioning). Next, we used a serial of cellular and animal validation assay to find key functional targets contributing to tumorigenesis (**F**unctional verification). Finally, we further revealed critical elements triggering alteration of specific chromatin sites and aberrant transcription of functional targets (**R**egulatory mechanism). To test our above hypothesis, we used and analyzed the dataset of ATAC-seq (GEO: GSE162380), H3K27me3 ChIP-seq (GEO: SRP312409) and RNA-seq (GEO: GSE194116) between human esophageal epithelial cell and esophageal squamous cell carcinoma (ESCC). In esophageal epithelial cell, we observed an open chromosomal region, chr11p15.4, which encodes the olfactory receptor protein OR51B5. However, we found that the *OR51B5* promoter region was closed in ESCC (Fig. 1B). In addition, we also observed that H3K27me3 was enriched in the *OR51B5* promoter region in ESCC (Fig. 1C). RNA-seq analysis further revealed that *OR51B5* expression was reduced in ESCC (Fig. 1D). We then examined the chromatin status of the *OR51B5* locus by formaldehyde-assisted isolation of regulatory elements (FAIRE) assay in ESCC cells, including KYSE450 and KYSE510 **(**Fig. 1E**)**. We showed that the chromatin status of *OR51B5* promoter (chr11:5,505,652–5,507,152) was actually closed (Fig. 1F, middle; Fig. 1G, lanes 5–6) as compared with upstream control site *OR51B8P* and downstream control site *UBQLN3* in ESCC cells (Fig. 1F, left and right), while the *OR51B5* promoter remained in an open chromatin status human esophageal epithelial cell Ne-3 (Fig. 1F, cycle; Fig. 1G, lane 4). These data suggest that a closed chromatin locus was found at *OR51B5* promoter in ESCC cells.

### CTCF-EZH2 diminish the accessibility of RNA polymerase II to *OR51B5* core promoter

**Fig. 2:**
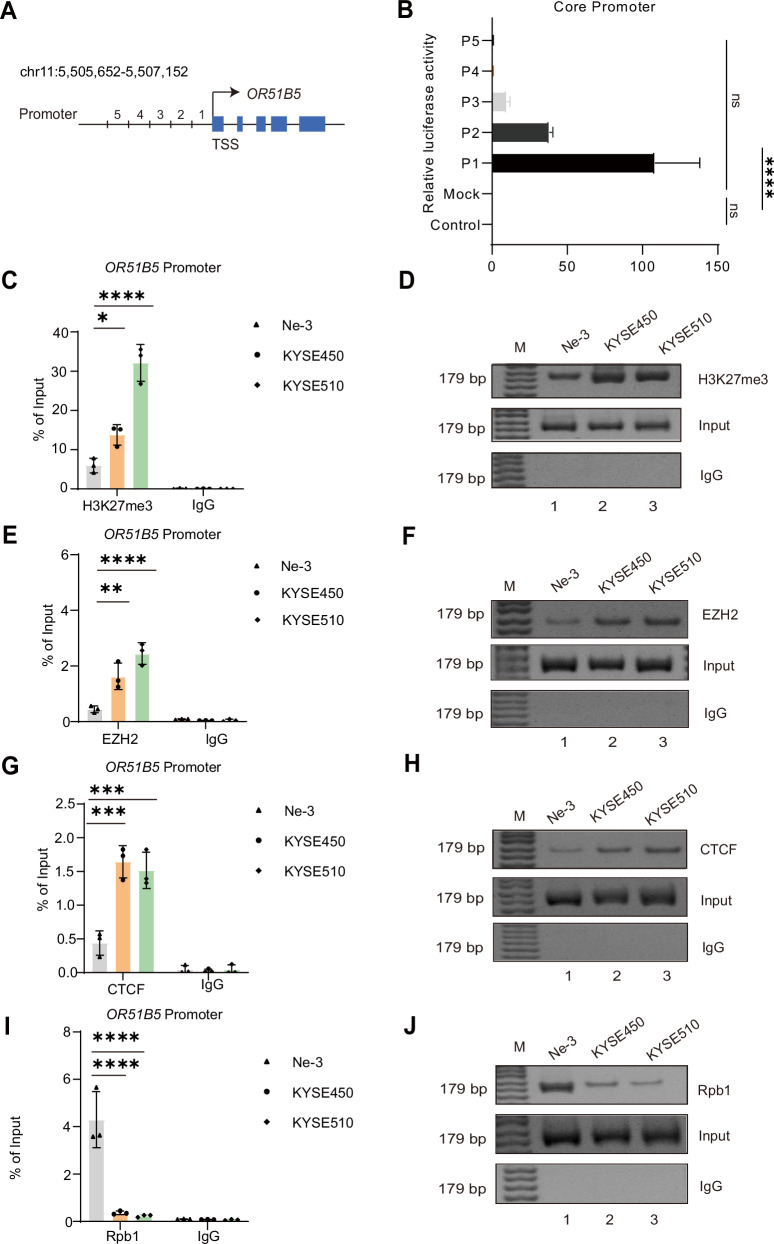
CTCF-EZH2 diminish the accessibility of RNA polymerase II to *OR51B5* core promoter. **A** Schematic diagram of the fragments of the *OR51B5* promoter. P1–P5: five fragments of the *OR51B5* promoter, TSS: transcriptional start site. **B** The luciferase activity of different fragments of *OR51B5* promoter. Ctrl, control group without any Firefly reporter plasmids; Mock, group with wild-type Firefly reporter plasmids; All groups had Renilla reporter plasmids. All data were calculated the ratio of firefly to Renilla luciferase activity (Fluc/Rluc) in dual luciferase reporter system. For comparison, the ratio of Fluc/Rluc of the mock was arbitrarily set as 1 in the calculation. All the experiments were performed in triplicate and data are presented as the mean ± SD using an unpaired two-tailed t test; ****P < 0.0001. **C** ChIP analysis of H3K27me3 to the *OR51B5* promoter in Ne-3, KYSE450 and KYSE510 cells. IgG served as the negative control. ChIP enrichment was presented as the percentage of input signal. Data are presented as mean ± SD from three independent experiments using a Dunnett’s multiple comparisons test; *P < 0.05; ****P < 0.0001. **D** PCR of ChIP assay of H3K27me3 at *OR51B5* core promoter region. Representative images from three independent experiments. Real-time ChIP-PCR assay of the EZH2 (**E**), CTCF (**G**) and Rpb1 (**I**) at the *OR51B5* promoter (P1 site) in Ne-3, KYSE450 and KYSE510 cells. Rabbit normal IgG served as the negative control. ChIP enrichment was presented as the percentage of input signal. Data are presented as mean ± SD from three independent experiments using a Dunnett’s multiple comparisons test; **P < 0.01; ***P < 0.001; ****P < 0.0001. PCR of ChIP analysis of EZH2 (**F**), CTCF (**H**) and Rpb1 (**J**) at the *OR51B5* core promoter in Ne-3, KYSE450 and KYSE510 cells.Representative images from three independent experiments.

To further test the altered chromatin status at *OR51B5* promoter, we examined the core promoter of *OR51B5*. We then set five 300 bp fragments covering promoter region of *OR51B5* (Fig. 2A, P1–P5). The dual luciferase reporter gene assay showed that the P1 fragment had the highest luciferase activity compared to control, mock and the other fragments (Fig. 2B), suggesting that the core promoter of *OR51B5* is located in the P1 region. Building on our previous H3K27me3 ChIP-seq results, we further investigated the chromatin modification status in the *OR51B5* promoter region to confirm the presence of H3K27me3 enrichment. Next, we detected the H3K27 trimethylation enrichment in the *OR51B5* promoter region by ChIP-qPCR assay, H3K27me3 level at the *OR51B5* promoter were significantly elevated in ESCC cells compared to Ne-3 cell (Fig. 2C, cycle and diamond**;** Fig. 2D, lanes 2–3). We further examined whether Enhancer of zeste homolog 2 (EZH2), the core component of Polycomb repressive complex 2 (PRC2), can bind to the *OR51B5* P1 promoter and has been shown to control the level of H3K27me3 [24]. ChIP-qPCR showed that enhancer of EZH2 was recruited to the *OR51B5* P1 promoter in both KYSE450 and KYSE510 cells (Fig. 2E, cycle and diamond; Fig. 2F, upper lanes 2–3). Because PRC2 easily interacts with CCCTC-binding factor (CTCF), we detected CTCF binding to the *OR51B5* P1 promoter in both ESCC and human esophageal epithelial cell. We showed that CTCF binding at the *OR51B5* P1 promoter were increased in ESCC cells (Fig. 2G, cycle and diamond; Fig. 2H, lanes 2–3) as compared to human esophageal epithelial cell (Fig. 2G, triangle). We next examined whether the accessibility of RNA polymerase II was altered due to the formation of repressive chromatin status at *OR51B5* P1 promoter. As the important subunit of RNA polymerase II in controlling gene transcription and elongation, RNA polymerase II maximal subunit B1 (Rpb1) was then chose in this assay. The results showed that Rpb1 was significantly diminish at *OR51B5* P1 promoter in ESCC cells (Fig. 2I, cycle and diamond; Fig. 2J, lanes 2–3) compared to Ne-3 cell (Fig. 2I, triangle). Collectively, these data suggest that increased binding of the CTCF-EZH2 diminish the accessibility of RNA polymerase II and promotes a repressive chromatin status at *OR51B5* core promoter [25].

### Closed chromatin status leads to inactivation of OR51B5 in ESCC

**Fig. 3:**
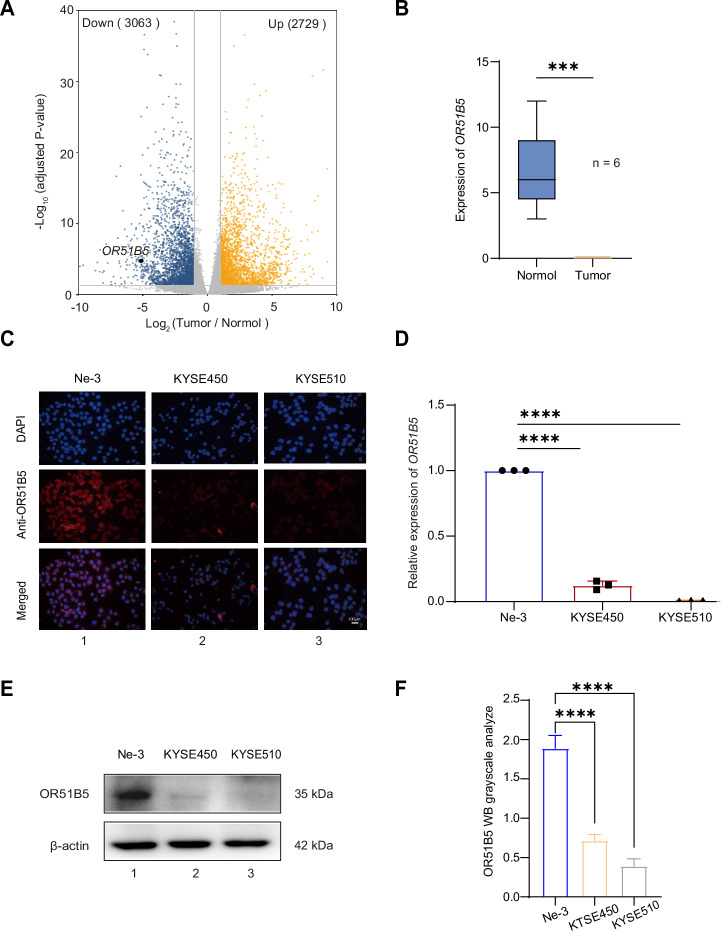
Closed chromatin status leads to inactivation of *OR51B5* in ESCC. **A** Volcano plot of differentially expressed genes in ESCC tumor samples compared with normal tissue samples. orange, up-regulated genes; blue, down-regulated genes; gray,unchanged genes (*n* = 6). **B** Combining tumor samples and normal samples from the GEO (GSE194116 dataset), *OR51B5* expression was significantly lower in ESCC tumor samples than in normal tissue samples. **C** Immunofluorescence of OR51B5 in Ne-3, KYSE450 and KYSE510 cells. Scale bar, 100 μm. **D** Real-time of *OR51B5* expression at RNA level in Ne-3, KYSE450 and KYSE510 cells. All the experiments were performed in triplicate and data are presented as the mean ± SD using an unpaired two-tailed t test; ****P < 0.0001. **E** Western blot of OR51B5 expression in human esophageal epithelial cell and ESCC cells. The protein ladder concentration was 20 μg. β-actin was used as negative control. Representative blots from three independent experiments. **F** Image J Statistical protein gray values, homogenized for OR51B5/β-actin values, relative gray value = gray value/homogenized value. Data are presented as mean ± SD from three independent experiments. Statistical significance was determined using a two-tailed *t* test. ****P < 0.0001.

We next examined whether closed chromatin status at *OR51B5* core promoter would inactivate the expression of *OR51B5*. By comparing the gene expression profiles between esophageal tumor and normal tissues, RNA-seq data (GEO: GSE194116) showed that 5792 differentially expressed genes were identified, including 2729 up-regulated genes and 3063 down-regulated genes. Among them, *OR51B5* was significantly down-regulated in tumor tissues compared to normal tissues (Fig. 3A, with fold changes >2). Further analysis revealed that *OR51B5* was low expressed in esophageal tumor tissues (Fig. 3B). By further visualization analysis, we observed that in normal esophageal tissue, there were expression peaks in five exons of *OR51B5* (Fig. S1A). To validate the above bioinformatics analysis, we then detected *OR51B5* expression in ESCC cells and Ne-3 cell. The immunofluorescence assay showed that OR51B5 was weakly expressed in KYSE450 and KYSE510 cells (Fig. 3C, lanes 2–3) as compared with control Ne-3 cell (Fig. 3C, lane 1). Next, we measured the expression of *OR51B5* in tumor cells using real-time PCR and western blot assay. As expected, we found that *OR51B5* was lower expressed in ESCC cells as compared to Ne-3 cell (Fig. 3D, square and triangle). To better reflect intracellular protein levels, we performed a protein concentration gradient experiment and determined that 20 µg was the OR51B5 optimal protein concentration for accurate detection (Fig. 3E and Fig. S1B–C). Western blot assay and quantitative densitometric analysis showed that OR51B5 was also low expressed in KYSE450 and KYSE510 cells (Fig. 3E, lanes 2–3; Fig. 3F, orange and gray). These data suggest that closed chromatin status at *OR51B5* promoter inhibits *OR51B5* transcription in ESCC.

### OR51B5 is a novel tumor suppressor in ESCC

**Fig. 4:**
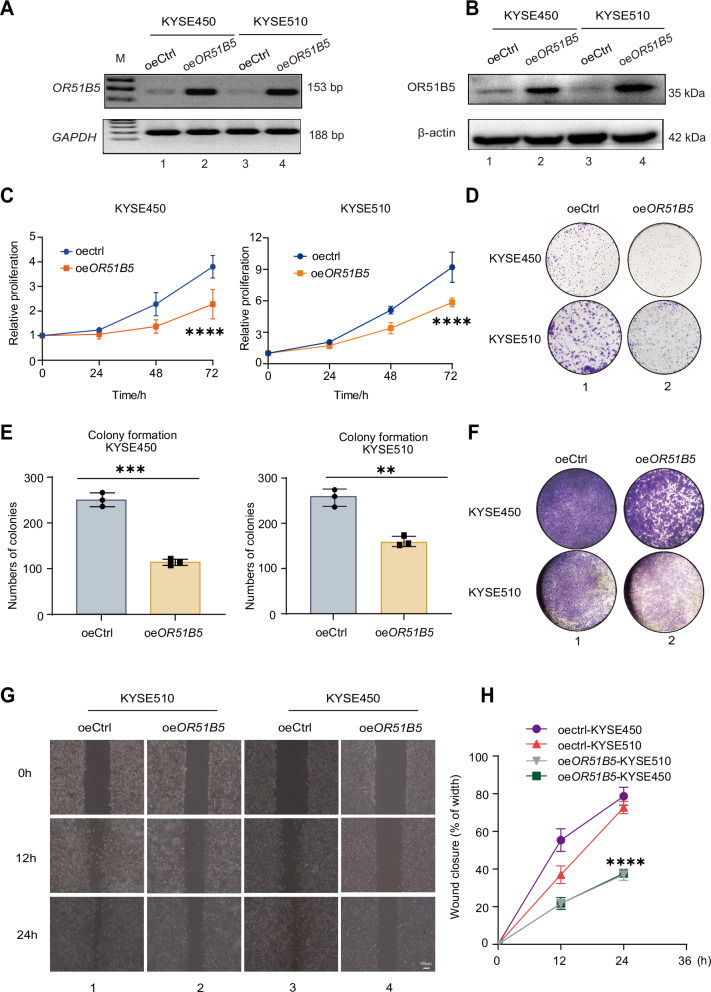
OR51B5 is a novel tumor suppressor in ESCC. **A** PCR of *OR51B5* overexpression in ESCC cells. *GAPDH* was served as a positive control. Representative images from three independent experiments. **B** OR51B5 expression was measured by western blot assay in *OR51B5*-overexpressing cells (*n* = 3). oeCtrl: pCDH-CMV empty vector, oe*OR51B5*: *OR51B5* overexpression. β-actin was used as the internal control. Representative blots from three independent experiments. **C** CCK-8 assay to measure 72 h cell growth rate in KYSE450 (left) and KYSE510 (right) cells after overexpressing *OR51B5*. Triplicate experiments were performed. Data are presented as the mean ± SD using a two-tailed Pearson’s r test; ****P < 0.0001. **D** Colony formation assay to determine the proliferative ability of ESCC cells after *OR51B5* overexpression. Representative images from three independent experiments. **E** Colony numbers were determined from three independent clone formation plates. Triplicates were performed. Data are presented as the mean ± SD, and the differences between two groups were calculated by unpaired two-tailed Student’s *t* test. **P < 0.01; ***P < 0.001. **F** Transwell migration assay to assess the migration ability of ESCC cells after *OR51B5*-overexpressing. Representative images from three independent experiments. **G** Representative images of the wound healing assay at 0-, 12- and 24-h post-scratch in oeCtrl and *OR51B5*-OE ESCC cells. Representative images from three independent experiments. scale bar: 100 μm. **H** Quantification of the migration distance. The migration distance was calculated as the percentage of the wound distance covered by cells at 24 h and 12 h compared to the initial wound distance at 0 h. Data are presented as mean ± SD from three independent experiments using an unpaired two-tailed *t* test; ****P < 0.0001.

To determine whether OR51B5 could alter tumor behavior, we overexpressed *OR51B5* in ESCC cells. PCR (Fig. 4A, lanes 2 and 4) and western blot assay (Fig. 4B, lanes 2 and 4) showed that *OR51B5* was successfully overexpressed in KYSE450 and KYSE510 cells. Next, we examined cell proliferation and colony forming ability in vitro. The proliferation of tumor cells was significantly reduced after overexpression of *OR51B5* compared to the control (oeCtrl) group (Fig. 4C, square). Subsequently, we performed an in vitro cell colony formation assay. We showed that the cell colonies were significant reduced in *OR51B5-*overexpressed ESCC cells (Fig. 4D, lane 2). The count numbers of cell colonies were reduced 55% and 39% (Fig. 4E, square) in *OR51B5-*overexpressed KYSE450 and KYSE510 cells. Similarly, transwell assays showed significant weak ability of cell migration in *OR51B5-*overexpressed ESCC cells (Fig. 4F, lane 2). Cell migration was further assessed through wound healing assays. We showed a two-fold reduction in migration capacity compared to the control group (Fig. 4G, lanes 2 and 4, Fig. 4H, inverted triangle and square). These data suggest that OR51B5 functions as a novel tumor suppressor in ESCC.

### Overexpression of *OR51B5* decreases tumor growth and metastasis in vivo

**Fig. 5:**
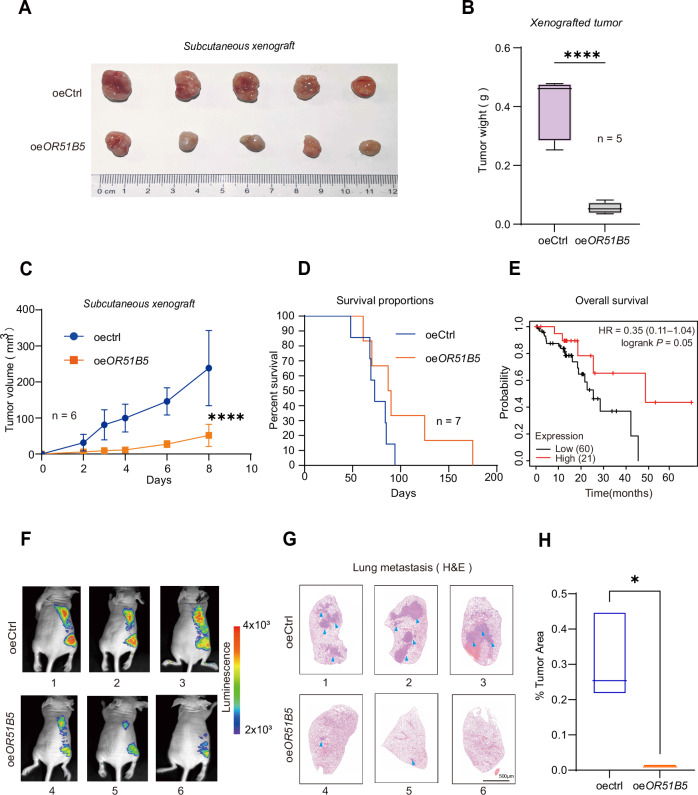
Overexpression of *OR51B5* inhibits the tumorigenesis of ESCC in vivo. **A** Photograph of orthotopic xenograft at 8 days after the subcutaneous injection of oeCtrl and oe*OR51B5* cells (*n* = 5 mice in each group). **B** Bar graph showed tumor weight (g) formed by the oeCtrl and oe*OR51B5* cells in a subcutaneous xenograft model. Tumor weight (g) was measured and presented as the mean ± SD (*n* = 5 mice) using an unpaired two-tailed *t* test; ****P < 0.0001. **C** A xenograft in vivo assay model presented tumor volume (mm^3^) formed by the oeCtrl and oe*OR51B5* cells. Tumor sizes (mm^3^) were calculated as the (length × width × width) × 0.52 and presented as the mean ± SD (*n* = 6 mice) using a Pearson’s r test, ****P < 0.0001. **D** Survival curve analysis of mice injected intravenously with oeCtrl and oeOR51B5 cells. The data are presented as the mean ± SD (*n* = 7) with two independent experiments. **E** Survival curves of ESCC patients with high and low *OR51B5* expression (https://kmplot.com/). **F** In vivo total-body bioluminescence images of athymic nude mice in dorsal positions (IVIS@ Imaging System) injected intravenously with oeCtrl or oe*OR51B5* cells, oeCtrl indicates cells express pCDH-CMV plasmid. oe*OR51B5* indicates cells overexpressing *OR51B5* (*n* = 3). **G** Representative images of the histological analysis of lung seeding in mice injected intravenously with oeCtrl or oe*OR51B5* cells (scale bar: 500 μm). **H** Quantification of the lung nodule area. Quantitative area ratios were calculated as the percentage of the stained area of the lung nodule to the mean of the total lung area. Data are presented as mean ± SD from three independent experiments using an unpaired two-tailed *t* test; *P < 0.05.

To investigate the contribution of the OR51B5 in tumor characteristics in vivo, *OR51B5*-overexpressed KYSE510 cells and control KYSE510 cells were injected subcutaneously into nude mice. We measured the size of the resultant tumors every 2 days for 8 days in subcutaneous xenografts. After 8 days, the mice were sacrificed, and the tumors were collected for further analysis (Fig. 5A). In the *OR51B5*-overexpressing groups, we found that both tumor weight (Fig. 5B, *n* = 5, *P* < 0.0001) and volume (Fig. 5C, *n* = 6, *P* < 0.0001) were significantly reduced compared with the control group. Similarly, the survival rate of *OR51B5*-overexpressing group was significantly higher than that of the control group (Fig. 5D, *n* = 7). In addition, TCGA showed that patients with low *OR51B5* expression had significantly shorter median survival times than patients with high *OR51B5* expression (Fig. 5E). To examine the tumor growth and migration capability after *OR51B5* overexpression in vivo, we establish metastatic models in nude mice. We then performed tail vein injection in nude mice and investigated systemic metastasis by bioluminescence and fluorescence imaging. The results showed that the luciferase signaling was significantly reduced at both proximal (lungs) and distal sites (bones) in the *OR51B5*-overexpressing group (Fig. 5F, bottom 4–6). After 8 weeks, the mice were euthanatized, and the organs were collected for further analysis. Lung metastasis often occurs in esophageal cancer [26]. Histological examination of lung tissue samples collected showed that overexpression of *OR51B5* significantly inhibited lung metastasis compared with control group (Fig. 5G, bottom row). Reducing the number and size of macroscopic nodules observed in the lungs (Fig. 5H). In conclusion, these studies suggest that OR51B5 plays a critical anticarcinogenic role in the growth and metastasis of ESCC in vivo.

### *OR51B5* activates Ca^2+^ signaling and inhibits N-Ras expression

**Fig. 6:**
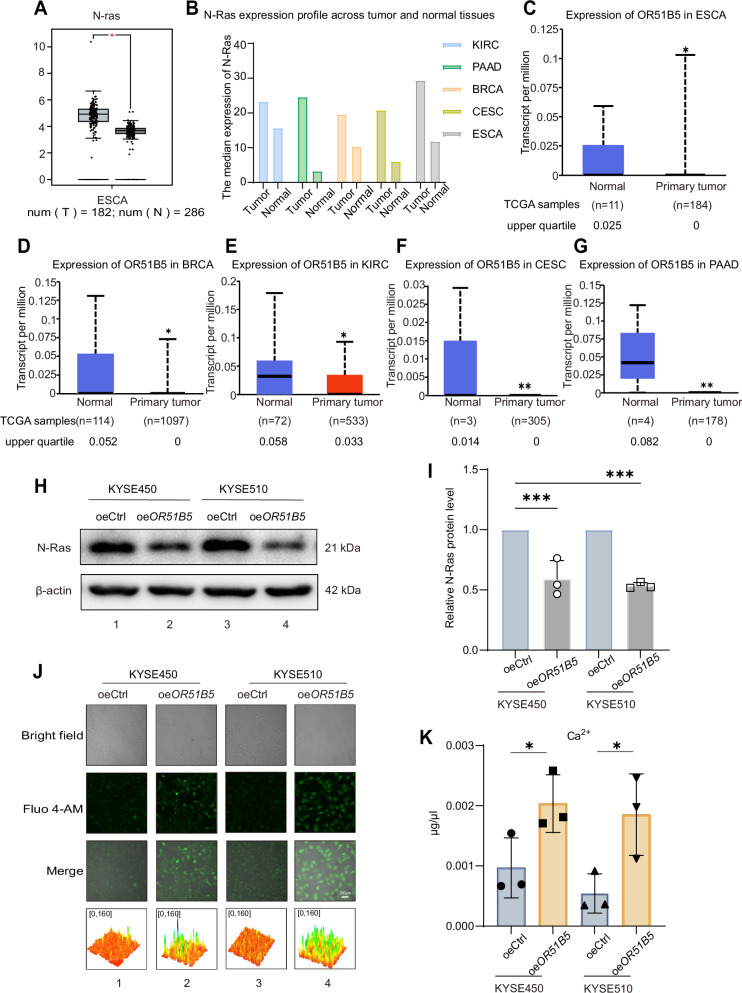
OR51B5 activates Ca^2+^signaling and inhibits N-Ras expresion. Gene expression profile (**A**) and boxplot (**B**) of N-Ras expression in public dataset from GEPIA (http://gepia.cancer-pku.cn/). T, tumor; N, normal. |Log2FC| cutoff was 1 and q-value cutoff was 0.01. Jitter size was 0.4. Log2 (TPM + 1) was used for log-scale. **C** Expression of *OR51B5* in esophageal carcinoma (ESCA)based on TCGA types from UALCAN. **D** Expression of *OR51B5* in breast invasive carcinoma (BRCA) based on TCGA types. **E** Expression of *OR51B5* in kidney renal clear cell carcinoma (KIRC) based on TCGA types. **F** Expression of *OR51B5* in cervical squamous cell carcinoma (CESC) based on TCGA types. **G** Expression of *OR51B5* in pancreatic adenocarcinoma (PAAD) based on TCGA types. **H** Western blot analysis showed that N-Ras expression levels in esophageal cancer cells with or without *OR51B5* overexpression. **I** Protein gray value statistics, β-actin normalised to control 1, were compared again. Date are presented as mean ± SD from three independent experiments. Statistical significance was determined using a two-tailed t test.***P < 0.001. **J** Ca^2+^ concentration was determined using Fluo 4-AM (Scale bar: 200 µm). Compared the luminance of the fluorescence maps using the 3D surface plot of ImageJ. **K** Ca^2+^ concentration was determined using calcium ion assay kit. The data are presented as the mean ± SD (*n* = 3). Statistical significance was determined using a two-tailed *t* test.*P < 0.05.

To further investigate which factors are regulated by OR51B5 in suppressing ESCC progression, we analyzed the G protein-coupled receptor—related signaling pathways. RAS, a classical proto-oncogene, is regulated by GPCR and is involved in the pathogenesis of many cancers. Furthermore, the expression level of N-Ras was significantly higher in some tumor samples, including esophageal cancer, compared with the corresponding normal tissues (Fig. 6A, B). Conversely, OR51B5 expression was found to be low in tumor samples and high in normal tissues. (Fig. 6C–G). To further investigate which factors was regulated by OR51B5 in inhibiting the proliferation and metastasis of ESCC cells, western blot assay and quantitative densitometric analysis confirmed that N-Ras isoform was significantly suppressed in *OR51B5*-overexpressing ESCC cells (Fig. 6H, lanes 2 and 4; Fig. 6I, right cycle and square). Additionally, we observed that N-Ras protein levels in human esophageal epithelial cell were slightly higher than those in tumor variant lines expressing ectopic OR51B5 (Fig. S2A, lanes 2–3). Building on the classical RAS pathway in KEGG and the potential involvement of Ca^2+^, we used Fluo 4-AM as a fluorescent probe to detect changes of Ca^2+^ concentration in the *OR51B5*-overexpressing cells. Laser confocal photography and 3D surface plot showed that significantly enhanced fluorescence was observed in the *OR51B5*-overexpressing cells as compared with the control group (Fig. 6J, lanes 2 and 4). In addition, a calcium content chromogenic assay showed that the Ca^2+^ concentration on content was increased in ESCC cells overexpressing *OR51B5* (Fig. 6K, square and inverted triangle). These data demonstrates that OR51B5 could increase intracellular the Ca^2+^ signal and diminished N-Ras expression during ESCC progression.

### Inhibition of N-Ras diminishes tumor cell proliferation and migration

**Fig. 7:**
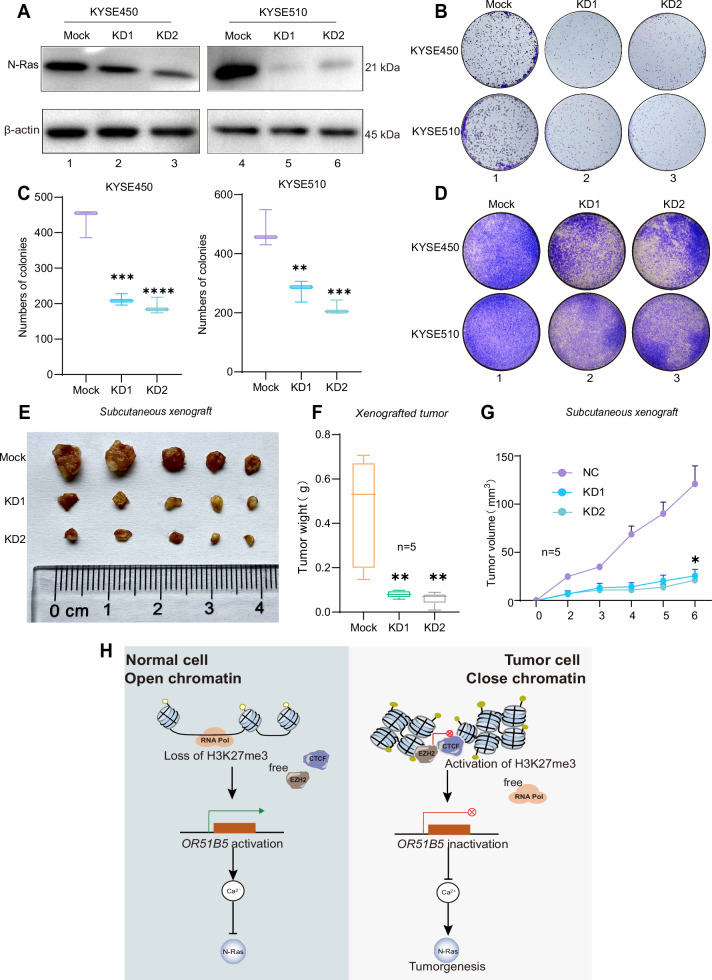
Inhibition of N-Ras diminishes tumor cell proliferation and migration. **A** Western blot of the expression of N-Ras at the protein level in esophageal cancer cells after N-Ras knockdown. Representative blots from three independent experiments. **B** Cloning formation assay assessed the colony formation ability of N-Ras knockdown in KYSE450 and KYSE510 cells; Representative images from three independent experiments. **C** Colony numbers were determined from three independent clone formation plates. Triplicates were performed. Data are presented as the mean ± SD, and the differences between two groups were calculated by unpaired two-tailed Student’s *t* test. **P < 0.01; ***P < 0.001; ****P < 0.0001. **D** Transwell migration assay to assess the migration ability of ESCC cells after N-Ras knockdown. Representative images from three independent experiments. **E** Photograph of orthotopic xenograft at 6 days after the subcutaneous injection of KYSE510 cells with or without N-Ras expression (*n* = 5 mice in each group). **F** Tumor weight (g) formed by the KYSE510 cells with or without N-Ras expression in a subcutaneous xenograft model. Tumor weight (g) was measured and presented as the mean ± SD (*n* = 5 mice) using an unpaired two-tailed *t* test; **P < 0.01. **G** A xenograft in vivo assay model presented tumor volume (mm^3^) formed by the KYSE450 cells with or without N-Ras expression. Tumor sizes (mm^3^) were calculated as the (length × width × width) × 0.52 and presented as the mean ± SD (*n* = 5 mice) using a Pearson’s r test. *P < 0.05. **H** In normal esophageal epithelial cell, the promoter of tumor suppressor *OR51B5* remains open chromatin status for activating *OR51B5* transcription and inhibits *N-Ras* expression. Once CTCF-EZH2 induces the close and repressive chromatin status by activating H3K27me3 and impaired the entry of RNA polymerase II at the *OR51B5* promoter, the transcription of *OR51B5* is inhibited and subsequently results in inactivation of Ca^2+^ signaling and activation of *N-Ras* expression for inducing esophageal carcinogenesis.

To investigate the oncogenetic role of N-Ras in esophageal cancer, we used short hairpin RNA (shRNA) to knockdown (KD) the expression of N-Ras in KYSE450 and KYSE510 cells. Western blot analysis showed that protein level of N-Ras were significantly reduced after transfecting with N-Ras shRNAs in ESCC cells (Fig. 7A, lanes 2–3 and 5–6). We next evaluated the effect of N-Ras knockdown on cell proliferation in esophageal cancer by performing a colony formation assay in vitro. We demonstrated a significant reduction in the growth rate of tumor cells following N-Ras knockdown in ESCC cells (Fig. 7B, lanes 2–3) as compared to the mock group (Fig. 7B, lane 1). The number of efficient colonies in the N-Ras silencing ESCC cells were only 44–57% (Fig. 7C, blue and green) of that observed in the mock group (Fig. 7C, purple).To further assess the potential role of N-Ras in promoting esophageal cancer tumor metastasis, we used the transwell assay, which demonstrated that silencing N-Ras significantly impaired the metastatic potential of esophageal cancer cells (Fig. 7D, lanes 2–3). To investigate the contribution of the N-Ras in tumor characteristics in vivo, we conducted animal experiments using a subcutaneous xenograft model in nude mice. The mice were injected with mock group and two N-Ras KD groups (KD1 and KD2) in the left underarm, with 2 million tumor cells per mouse. After 6 days, the mice were euthanatized, and the tumors were collected for further analysis (Fig. 7E, *n* = 5). As expected, tumor weight (Fig. 7F, *n* = 5, *P* < 0.001) and volume (Fig. 7G, *n* = 5, *P* < 0.05) were notably reduced in the N-Ras knockdown group. In addition, in order to further investigate the regulatory mechanisms of N-Ras in a non-cancer environment, we successfully knocked down N-Ras in Ne-3 cell (Figure S2B, lanes 2–3). Cell colony formation assay showed that the growth rate of normal esophageal cells was not significantly reduced after N-Ras knockdown, and effective colony counts were 85-88% of the mock group (Figure S2C, lanes 2–3; Fig. S2D, square and triangle). Similarly, transwell experiments showed weak inhibition of metastasis in esophageal epithelial cells by knockdown of N-Ras compared to the mock group (Fig. S2E, lanes 2–3). Thus, these results suggest that N-Ras plays an oncogene role in esophageal cancer.

## Discussion

Aberrant OR signaling is intricately linked to the progression of malignant tumors [27, 28]. ORs regulate cellular signaling pathways, oncogene expression, and interactions with the tumor microenvironment (TME), thereby contributing to tumorigenesis [29, 30]. Although the dysregulation of *OR* gene expression has been reported in various cancers, its mechanistic role and functional significance in tumorigenesis remain incompletely understood. In this study, we successfully established a CiFR workflow and identified the OR51B5 served as novel tumor suppressor in esophageal cancer progression. We further found that CTCF-EZH2 contributes to the repressive chromatin status on the *OR51B5* promoter and impaired the entry of RNA polymerase II, subsequently inactivating *OR51B5* to activate N-Ras expression and promote the growth and metastasis of esophageal cancer (Fig. 7H).

It should be noted that a plenty of functional chromatin sites remain undiscovered during tumorigenesis, and the development of new therapeutic targets requires the identification of more efficient chromatin sites [31–33]. Many studies have used ATAC-seq to detect chromatin opening status across the genome [34, 35]. In addition, the chromatin state of specific loci can be determined by identifying specific protein-DNA interactions using ChIP-seq [36]. In this study, we present an alternative comprehensive workflow that combines multiple high-throughput sequencing techniques and tumor functional analysis to efficient reveal the functional chromatin locus that triggers tumorigenesis. Since a number of targets are regulated by dynamic chromatin, it is likely to provide an alternative choice and would be great interesting to further identify functional targets by using CiFR workflow in various physiological and pathological processes.

Olfactory receptor transcription is influenced by various factors, including the expression of a single allele and the presence of specific transcriptional binding sequences in the promoter region [20, 37]. Recent studies have highlighted the crucial role of inhibitory chromatin in the regulation of olfactory receptor expression in mammals [38, 39]. Single-cell chromosome conformation capture (3 C) have demonstrated that olfactory sensory neurons (OSNs) establish robust and specific inter-chromosomal interactions among odorant receptor gene clusters, which undergo heterochromatic modifications to ensure the complete silencing of *OR* genes [40, 41]. Within the main olfactory epithelium (MOE), heterochromatin compacts and silences olfactory receptor genes [42]. Nevertheless, it remains unclear whether alterations in chromatin dynamics exert regulatory control over the transcription of olfactory receptor genes, particularly in relation to the triggering of tumorigenesis. It should also be noted that *OR51B5* is located on human chromosome 11 at locus 11p15.4, with a full-length 1454 bp gene encoding a 35 kD protein product [6]. Previous studies have shown that *OR51B5* ligand isononyl alcohol increased the levels of intracellular Ca^2+^ in both AML patient blood cells and K562 cells, activates *OR51B5* expression and inhibits cell proliferation [43]. UVB irradiation or dexamethasone also could induced inhibition of *OR51B5* expression [44]. In this study, we firstly found that increased aberrant CTCF-EZH2 binding reduced Rpb1 enrichment and increased H3K27me3 at the *OR51B5* promoter region, leading to chromatin closure and repression of *OR51B5* expression. Although we cannot theoretically rule out other factors involved in *OR51B5* transcriptional regulation, this study is the first to suggest a potential association between chromatin modifications and *OR51B5* expression. The potential effect of other chromatin regulatory factors on *OR51B5* transcription, whether activating or suppressive, cannot be theoretically excluded. Therefore, it will be interesting to focus on identifying other factors to better understand the transcriptional regulation of *OR51B5* and tumorigenesis.

ESCC is the sixth leading cause of cancer-related deaths worldwide, claiming the lives of more than 400,000 patients annually [45, 46]. In China and East Asia, ESCC is the most common pathologic histologic type of esophageal cancer [47]. Despite significant advances in diagnosis and treatment, the 5-years survival rate of ESCC patients remains below 20% [48, 49]. The development of ESCC is a multifactorial process [50]. Transcription factors TP63, SOX2, and KLF5 have been demonstrated to influence chromatin dynamics and contribute to cancer-specific gene *ALDH3A1* inactivation in ESCC [51]. It should also be demonstrated that olfactory receptors constituted the largest family of G protein-coupled receptors and were initially expressed only in olfactory tissues. However, ORs have recently been shown to be much more versatile than originally thought and have been implicated in the regulation of many physiological and pathological events outside the nasal epithelium [52–55]. OR2AT4 could regulate the growth of human hair growth while *Olfr2* could drive the production of atherosclerosis [56, 57]. Additionally, olfactory receptors have also shown important roles in tumorigenesis. OR5H2 could regulate endometrial cancer cell proliferation by interacting with the IGF1 signaling pathway [30], and OR5B21 drove breast cancer metastasis [28]. OR51B5 acted as a G protein-coupled receptor and is involved in G protein-mediated odor signaling, triggering neuronal responses and odor perception [58]. In this paper, however, we for the first time show that OR51B5 acts as a tumor suppressor in ESCC and is significantly associated with survival in ESCC patients. This finding provides a promising therapeutic target in the tumorigenesis of ESCC and helps predict patient survival and prognosis. We also demonstrated that activation of *OR51B5* expression increases intracellular Ca^2+^ signaling and reduces N-Ras expression for inhibiting ESCC growth and metastasis. Furthermore, we revealed the oncogenic role of N-Ras in esophageal cancer. Future studies will focus on exploring other potential oncogenic pathways and their interactions with N-Ras, which will provide a broader context for understanding the complex regulatory networks involved in tumorigenesis. In particular, understanding how Ca^2+^ modulate key signaling molecules like N-Ras will be essential for identifying novel therapeutic targets and strategies for cancer treatment. Additionally, inactivation of olfactory genes may lead to the dysregulation of above pathways, contributing to an environment that favors tumor development. These findings underscore the complex interplay between olfactory signaling and tumorigenesis, indicating that further research into the specific mechanisms by which olfactory receptors influence cancer biology is warranted.

## Materials and methods

All animal procedures involving xenograft model establishment and imaging were conducted in accordance with protocols approved by the Tongji University Experimental Animal Center. Five-week-old female BALB/c nude mice (GemPharmatech) were selected for the experiment, given that the gender of the patients did not exert a significant influence on the results or conclusions. The tumor size was meticulously monitored, ensuring that the maximum allowable diameter did not exceed 1 cm^3^. At the conclusion of the experiment, the mice were euthanized via cervical dislocation, and the tumors, along with the major organs, were harvested using surgical scissors.

### Cell culture and transfection

Human ESCC cell lines KYSE450, KYSE510 and the normal esophageal epithelial cell line Ne-3 were all maintained in RPMI-1640 medium (Gibco) with 10% fetal bovine serum (Gibco), 100 μg/mL streptomycin and 100 μg/mL penicillin. Cells were cultured in a 5% CO_2_ incubator at 37 °C.

### RNA extraction and RT-qPCR

RNA extraction was performed with TRIzol reagent (Sigma), and complementary DNA (cDNA) was generated using the 1st Strand cDNA Synthesis SuperMix (Yeasen, Shanghai). Transcript quantification was conducted via real-time PCR on a Roche LightCycler 96 system, employing SYBR Green Master Mix (Yeasen, Shanghai) for detection. The data represents the fold change (FC) of experimental group versus control group. △Ct = Ct (test gene) – Ct (reference gene). △△Ct =△Ct (experimental group) -△Ct (control group). The FC of a test gene in experimental group versus control group was calculated as FC = 2^-△△Ct^. Each gene tested in triplicates in every independent experiment, and all experiments were triplicated. Primers used are listed in Table S1.

### Luciferase assay

Genomic DNA (gDNA) was isolated using the Genomic DNA Extraction Kit according to the manufacturer’s instructions, followed by PCR amplification with Q5 High-Fidelity DNA Polymerase (NEB) using primers listed in Table S1. The promoter region upstream of *OR51B5* TSS was dissected into 5 fragments of 300 bp and were respectively cloned into the pGL3-basic vector (Promega). HEK-293T cells were seeded in 24-well plates and allowed to reach approximately 50% confluence after 24 h. Each well received a mixture of 900 ng of the constructed pGL3-basic plasmids (containing different *OR51B5* promoter fragments or the empty vector) along with 90 ng of the pRL-TK control plasmid (Promega), delivered using Lipofectamine 3000 (Invitrogen). Following a 48-h incubation, luciferase activity was assessed using the Dual Luciferase Reporter Gene Assay Kit (Yeasen, Shanghai) as per the provided protocol. Each group was repeated with three technical replicates. All experiments were performed in triplicate, with three independent biological replicates conducted. Firefly luciferase activity was normalized to Renilla luciferase activity. *P* values were calculated using test.

### Lentivirus packaging and generation of stable cell lines

The *OR51B5* fragment was introduced into the pCDH-CMV-MCS-EF1-Puro lentiviral vector (System Biosciences), respectively. The above vectors were incubated with lipofectamine 3000 (Invitrogen) with Opti-MEM Reduced Serum Medium (Gibco), 3 mg pMD2.D plasmid and 6 mg PsPax plasmid and transfected with HEK-293T cells. Viral supernatant was harvested at 48- and 72-h post-transduction, then filtered and concentrated. Prior to infection, esophageal cancer cells were plated in twelve-well plates at a density of 4.0 × 10^5^ cells per well and allowed to adhere for 24 h. The culture medium was then replaced with virus-laden supernatant containing 10 ng/ml polybrene (Sigma). Following a 48-h incubation, fresh medium was applied. To establish stable knockout lines, cells underwent puromycin selection (8 μg/ml, Invitrogen) for two weeks. Single-cell-derived colonies were isolated, and later expanded for further analyses.

### Tumor xenograft nude mice model

All animal maintenance and experimental procedures were performed according to the Tongji University Guide for the use of laboratory animals. 5 × 10^6^ cells (KYSE510^oeCtrl^ and KYSE510^oe*OR51B5*^) in 100 μl volume of DPBS were subcutaneously injected into the right anterior subcutaneous part of 5-week-old female nude mice. Tumor volume was monitored using a vernier caliper every 2 days and calculated with the formula: length (mm) × width (mm) × width (mm) × 0.52. Five mice from each group were sacrificed, and the tumors were weighed. A total of 2 × 10^6^ cells (KYSE510^Mock^, KYSE510^KD1^, KYSE510^KD2^) were subcutaneously injected into the right anterior subcutaneous part of 5-week-old female nude mice, with a volume of 100 μl of DPBS. The tumor volume was monitored using a vernier caliper at 1-day intervals and calculated with the formula: length (mm) × width (mm) × width (mm) × 0.52. Subsequent to the monitoring period, five mice from each group were euthanized, and the resulting tumors were weighed. All experimental procedures adhered to ethical regulations, with tumor sizes strictly maintained below 1 cm^3^.

### ChIP-Seq data processing

The Chromatin immunoprecipitation sequencing (ChIP-seq) publicly available data used in this study are available under SRA accession code SRP312409 [59]. The sequencing reads were aligned to the hg38 version of the human reference genome using Bowtie2 (version 2.3.4.1) [59]. Subsequently, SAM files were converted to BAM files and sorted using samtools (version 1.12) [60]. To avoid false estimation of gene expression, PCR duplicates were identified and removed from mapped reads using Sambamba (version 0.6.6) [61]. The coverage files were then converted to BigWig files for visualization using deeptools (version 2.5.7) [62].

### Tumor metastasis nude mice model

All animal maintenance and experimental procedures were performed according to the Tongji University Guide for the use of laboratory animals. Cells were transduced with lentiviral vector, followed by selection with 8 mg/ml G418 (InvivoGen) for two weeks. 1.5×10^6^ cells (KYSE450^oeCtrl^, KYSE450^oe*OR51B5*^) in 100 μl volume of DPBS were added to 200 μl of DPBS and injected into 5-week-old female nude mice through the tail vein. After 8 weeks, mice were intraperitoneally administered D-Luciferin Sodium Salt (Yeasen, Shanghai), and bioluminescence was monitored using an in vivo imaging system under anesthesia. Five mice in each group were executed, and three groups were selected for pathological sections.

### RNA extraction and quality control

Total RNA was isolated from samples, and its purity and concentration were verified using a 2100 Bioanalyzer (Agilent Technologies) and a Qubit 2.0 fluorometer with the Qubit RNA Assay Kit (Invitrogen). For subsequent experiments, 2 μg of high-quality total RNA per sample was prepared.

### Western blot assay

Cell lysis was performed using RIPA buffer (Yeasen, Shanghai) supplemented with 1 mM PMSF (Yeasen, Shanghai) for 30 min, followed by centrifugation at 13,000 × *g* (10 min, 4 °C). The obtained protein lysate was separated by 10% SDS-PAGE and electro transferred to PVDF membranes (Millipore). After 2 h blocking with 5% non-fat milk (Sangon Biotech) at room temperature, membranes were probed with primary antibodies in 5% TBST overnight at 4 °C. The membrane was then incubated with Peroxidase-Conjugated Goat Anti-Rabbit IgG (1:20000) or Peroxidase AffiniPure Goat Anti-Mouse IgG (1:10000). The band signals were visualized and quantified using the Fully Automatic Chemiluminescence/Fluorescence Image Analysis System (Tanon). The following antibodies were used in this study: anti-β-actin (1:1000, Yeasen, 30101ES50), anti-OR51B5 (1:1000, CUSABIO, CSB-PA629449, anti-N-Ras (1:1000, ABclonal, A7566)).

### Immunocytochemistry

For cell passaging culture, logarithmically grown cells were taken and cultured on glass bottom slides, and when the cells reached 80% growth, fixed with 4% paraformaldehyde for 15 min at 37 °C and incubated with 2.5% BSA for 60 min at RT. OR51B5 antibody (1:200, Thermo Fisher, PA5-10227) incubated overnight at 4 degrees. After having rewashed the cells three times with PBST, the cells were incubated with a secondary goat anti-rabbit antibody (1:1000) in blocking solution for 1 h at room temperature. Next, the cells were washed again with PBST and mounted on a slide with 5ul DAPI Staining Solution (beyotime, C1005).

### ChIP assay

ChIP was performed using an EZ-Magna ChIP A/G kit (Millipore) following the manufacturer protocol. In brief, 10^7^ cells were crosslinked and then lysed. DNA was sheared to a size of 200–500 bp (Covaris M220). Anti-CTCF, Anti-EZH2, Anti-H3K27me3 and Anti-Rpb1 used for ChIP. Anti-normal IgG was used as a negative control. After immunoprecipitation on A/G beads, DNA was purified and amplificated with qPCR. Primers for ChIP-qPCR are listed in Table S1. The following antibodies were used in this study: anti-CTCF (1:50, CST, 3418), anti-H3K27me3 (10 μg, ABclonal, A22006), anti-IgG (5 μg, ABclonal, AC005), anti-EZH2 (1:100, CST, 5246), anti-Rpb1 (1:100, CST, 2629).

### Transwell assay

The migration ability of the cells was evaluated by 24-well transwell chamber (Corning). 50,000 cells were suspended in 200 μl RPMI-1640 medium containing the basal membrane matrix (Corning) in the upper chamber and 600 μl RPMI-1640 medium with 20% FBS added in the lower chamber. After incubation at 37 °C for 48 h, it was dyed with 0.25% crystal violet. Scrub with water to remove the cells on the inside of the transwell and photograph the cells on the outside.

### Cell counting kit-8 (CCK-8) assay

For the CCK-8 assay, the transfected cells (1 × 10^3^ cells/well) were seeded in quintuplicate into 96-well plates and cultured to complete adherence. CCK-8 solution (10 μl, Yeasen, shanghai) was added to each well at 0, 24, 48 and 72 h. After 2 h of incubation, the absorbance was measured on a microplate reader at 450 nm in microplate reader (SpectraMax iD3).

### Colony formation assay

Transfected cells (1 × 10^3^ cells/ well) were transplanted into a 6-well plate for colony formation experiments. After 8 days, the cells were fixed with 70% methanol (Sigma) and stained with crystal violet (Beyotime) for 20 min. The number of effective colonies was manually counted under a microscope and the colony formation efficiency of the cells on each plate was calculated.

### Colorimetric assay of calcium content

For cell counting, an equal number of cells were seeded into a 6-well plate, removed after 12 h, the culture medium was removed and washed again with PBS. Add 120 µl of sample lysate to each well. Blow several times with a gun to make full contact between sample lysate and cells. After sufficient lysis, centrifuge at 4 °C 10,000–14,000 × *g* for 3–5 min, remove supernatant and place on ice for measurement. Prepare calcium standards by adding 50 µl of standard or sample per well to a 96-well plate. Subsequently, add 150 µl of working solution per well and mix well. Incubate for 5–10 min at room temperature and protected from light. Measure the absorbance at 575 nm with an enzyme marker and make a standard curve. Calculate the calcium ion concentration in the 6-well plate.

### Calcium imaging

Remove the pre-cultured cells, remove the medium and wash the cells 3 times with HBSS solution. Add Fluo-4, AM working solution to the cells in an amount that covers the cells. Incubate at 37 °C for 50 min, then remove Fluo 4-AM working solution. Cells were washed 3 times with HBSS solution to fully remove the residual Fluo 4-AM working solution. Then add HBSS solution to cover the cells. Incubate at 37 °C for about 30 min to ensure complete de-esterification of the AM moiety within the cells. Cells were examined by laser confocal or fluorescence microscopy with an excitation wavelength of 494 nm and an emission wavelength of 516 nm.

### Statistical analysis

Three independent biological replicates (n) were included in the analyses. Statistical evaluations were conducted using GraphPad 9.0 and Microsoft Excel. The determination of statistical significance followed the following criteria: *P < 0.05, **P < 0.01, ***P < 0.001, ****P < 0.0001, ns = not significant (*P* > 0.05). The data are presented as the mean ± standard deviation (SD). The source data can be found in the Source Data file.

## Supplementary information

figure S1

figure S2

Table S1

Supplemental Figure legends

Original western blots

## Data Availability

The data generated in this study are available upon request from the corresponding authors. TCGA patient samples analyzed here are available in the public data portal (https://kmplot.com/analysis/). Gene expression profile and boxplot of N-Ras expression in public dataset from GEPIA (http://gepia.cancer-pku.cn/). Gene expression profile and boxplot of OR51B5 expression in public dataset from UALCAN (https://ualcan.path.uab.edu/). The RNA-seq publicly available data used in this study are available under GEO (GSE194116). The ChIP-seq and ATAC-seq publicly available data used in this study are available under SRA accession code SRP312409 and GEO (GSE162380).
