# Supplementary figures and images for: Olfactory receptor OR51B5 suppressed esophageal cancer progression through activates Calcium / N-Ras signaling
Source: Cell Death Dis. 2025 Jun 16;16(1):450. doi: 10.1038/s41419-025-07769-9 (PMC12170851; doi:10.1038/s41419-025-07769-9)

**A**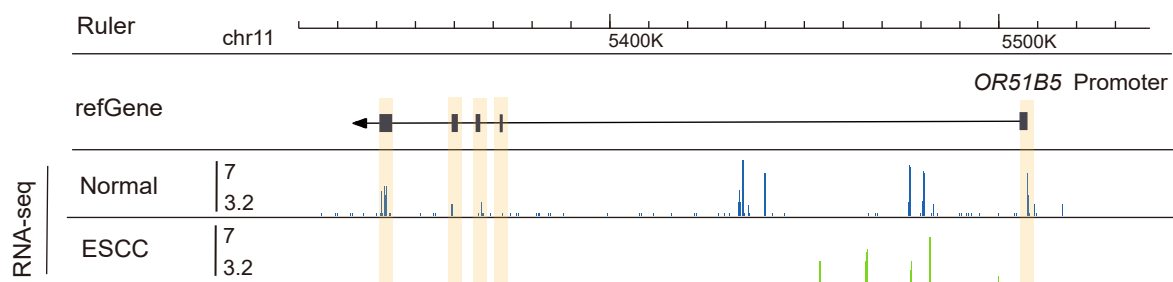**B**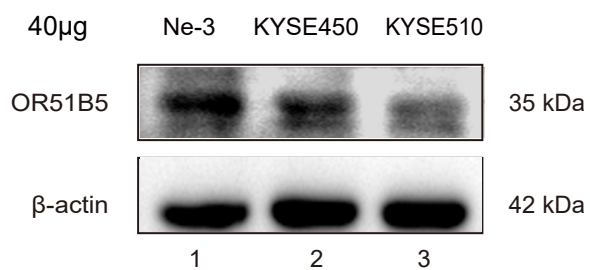**C**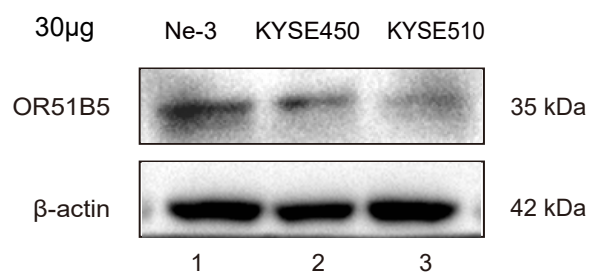

Supplement: Supplementary file 1 — figure S1 [file 41419_2025_7769_MOESM1_ESM.pdf]

**A**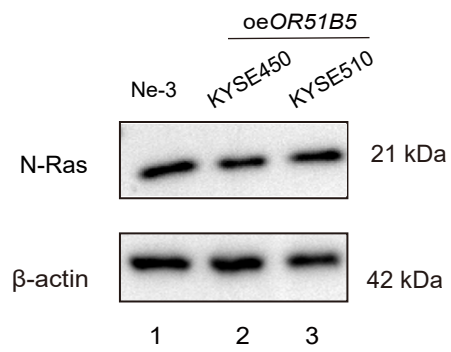**B**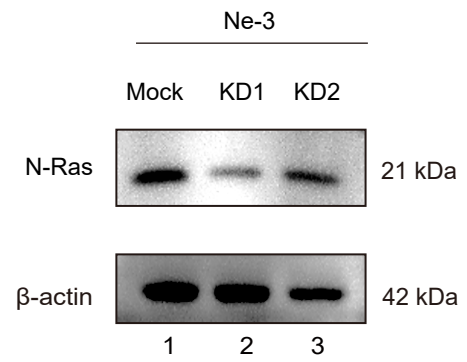**C**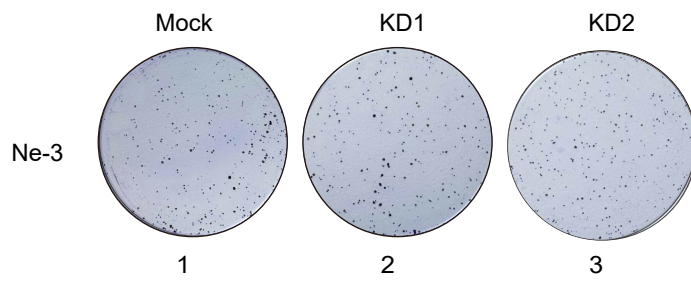**D**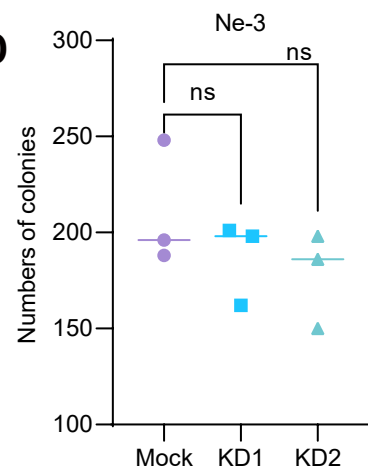**E**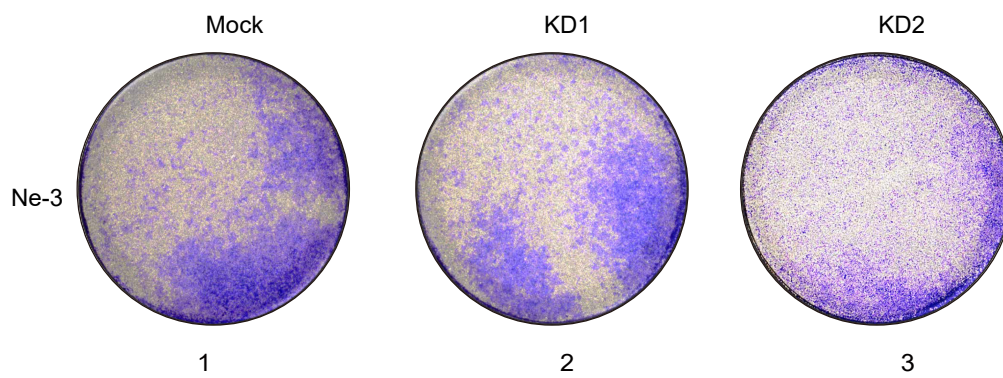

Supplement: Supplementary file 2 — figure S2 [file 41419_2025_7769_MOESM2_ESM.pdf]
