# Supplementary material for: Olfactory receptor OR51B5 suppressed esophageal cancer progression through activates Calcium / N-Ras signaling
Source: Cell Death Dis. 2025 Jun 16;16(1):450. doi: 10.1038/s41419-025-07769-9 (PMC12170851; doi:10.1038/s41419-025-07769-9)
Supplement: Supplementary file 3 — Table S1 [file 41419_2025_7769_MOESM3_ESM.docx]

Table S1. Primers used in this study.

| Primer name | Sequences (5’-3’) | Purpose |
| --- | --- | --- |
| *GAPDH*-F | GGGAGCCAAAAGGGTCATCA | RT-qPCR |
| *GAPDH*-R | TGGTCATGAGTCCTTCCACG | RT-qPCR |
| *OR51B5*-F | TTTTTGGCAATGGCACCCTC | RT-qPCR |
| *OR51B5*-R | TGTGATCCAGCCAGAGGACT | RT-qPCR |
| *OR51B5*-P-F | ATCTCGTGAGACCCATTCACTG | ChIP-qPCR |
| *OR51B5*-P-R | CCTGATATGATTTGGCTCTGTGGC | ChIP-qPCR |
| TSS-P1-F | CTCGGTACCGAGCTTGTGTAGAGCAAC | Luciferase assay |
| TSS-P1-R | GCAACGCGTGTGTAACTTAGAGCTGCT | Luciferase assay |
| TSS-P2-F | CTCGGTACCGTTACACAGGCTGATC | Luciferase assay |
| TSS-P2-R | GCTACGCGTCCACTGTAGAGAAACGC | Luciferase assay |
| TSS-P3-F | CACGGTACCTGGGTTTACCTGACAC | Luciferase assay |
| TSS-P3-R | GCAACGCGTGCTTCTGTGAGTGTGTAC | Luciferase assay |
| TSS-P4-F | GGAGGTACCGCCTTTCCTTTCTCC | Luciferase assay |
| TSS-P4-R | GCAACGCGTCTTTATACAGTCCCTTG | Luciferase assay |
| TSS-P5-F | GCTGGTACCTAAAGTCGGTAAGATTC | Luciferase assay |
| TSS-P5-R | GCTACGCGTCAGAGGAGGAAATGTTG | Luciferase assay |
| Sh-N-Ras-1 | CAGTGCCATGAGAGACCAATA | knockdown |
| Sh-N-Ras-2  Sh-N-Ras-3 | CAAGAGTTACGGGATTCCATT  GAAACCTGTTTGTTGGACATA | Knockdown  knockdown |
