# Supplementary material for: Olfactory receptor OR51B5 suppressed esophageal cancer progression through activates Calcium / N-Ras signaling
Source: Cell Death Dis. 2025 Jun 16;16(1):450. doi: 10.1038/s41419-025-07769-9 (PMC12170851; doi:10.1038/s41419-025-07769-9)
Supplement: Supplementary file 4 — Supplemental Figure legends [file 41419_2025_7769_MOESM4_ESM.docx]

**Supplemental information**

Supplementary files

Figure legends

Supplemental information includes 2 figures and 1 table.

Supplementary Figures

Supplementary Figure 1. The expression of *OR51B5.*

**A.** RNA-seq visualization analysis revealed that *OR51B5* exon peaks were enriched in normal esophageal tissue. **B-C.** Western blot of OR51B5 expression in Ne-3, KYSE450 and KYSE510 cells. Protein gradients were performed setting 40 μg,30 μg.

Supplementary Figure 2. N-Ras does not inhibit the growth and metastasis of Ne-3.

**A.** Western blot of the expression of N-Ras at the protein level in Ne-3 and *OR51B5*- overexpression cell. Representative blots from three independent experiments. **B.** Western blot of the expression of N-Ras at the protein level in Ne-3 cell after N-Ras knockdown. Representative blots from three independent experiments. **C.** Cloning formation assay assessed the colony formation ability of N-Ras knockdown in Ne-3 cell; Representative images from three independent experiments. **D.** Quantification of visible colonies with or without N-Ras in Ne-3 cell. Data are presented as the mean ± SD, and the differences between two groups were calculated by unpaired two-tailed Student’s t test. ns: no significant difference. **E.** Transwell migration assay to assess the migration ability of Ne-3 cell after N-Ras knockdown. Representative images from three independent experiments.
