# Supplementary material for: Olfactory receptor OR51B5 suppressed esophageal cancer progression through activates Calcium / N-Ras signaling
Source: Cell Death Dis. 2025 Jun 16;16(1):450. doi: 10.1038/s41419-025-07769-9 (PMC12170851; doi:10.1038/s41419-025-07769-9)
Supplement: Supplementary file 5 — Original western blots [file 41419_2025_7769_MOESM5_ESM.pdf]

Original western blots Figure 3E

Ne-3 KYSE450 KYSE510

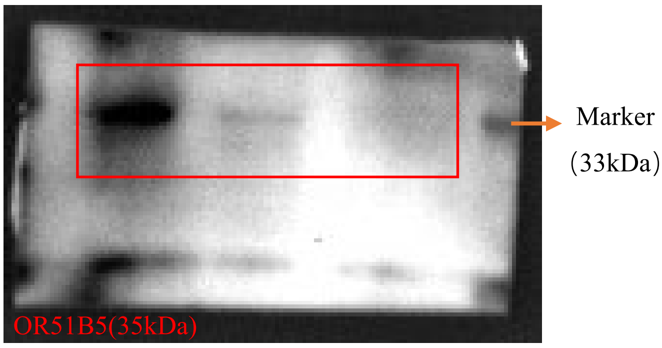

Marker
(33kDa)

Ne-3 KYSE450 KYSE510

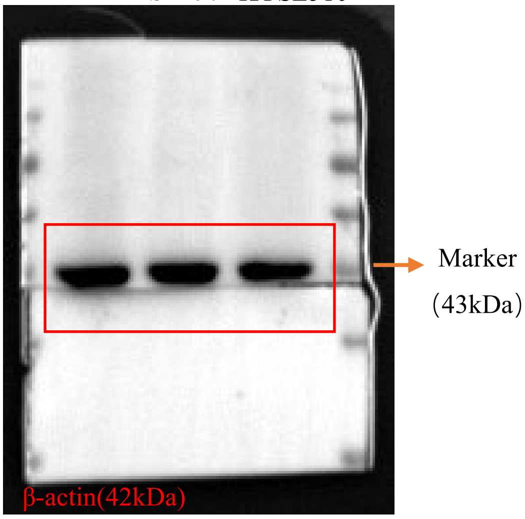

Marker
(43kDa)

Original western blots Figure 4B

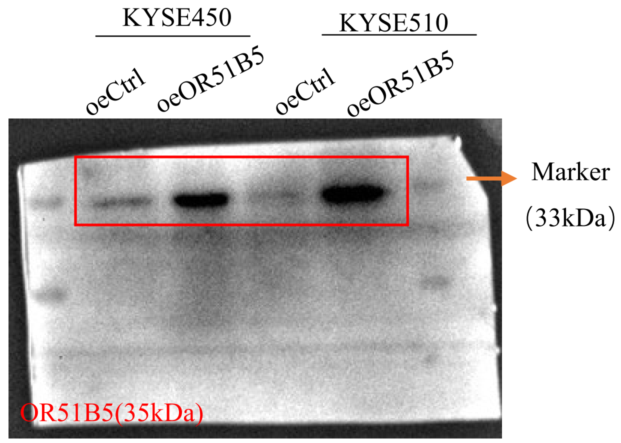

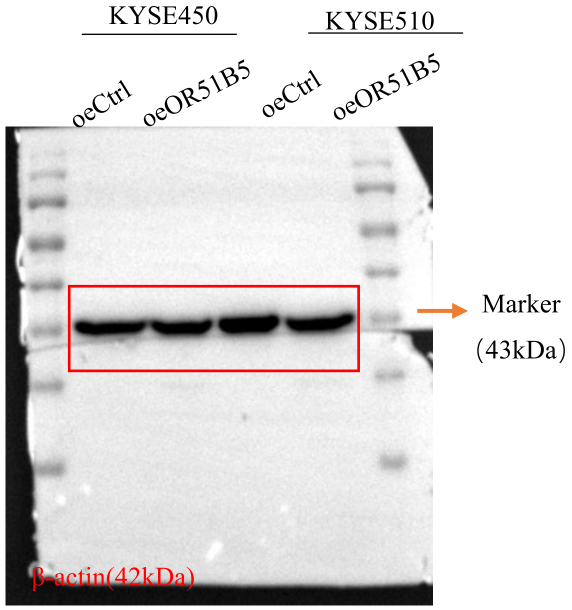

Original western blots Figure 6H

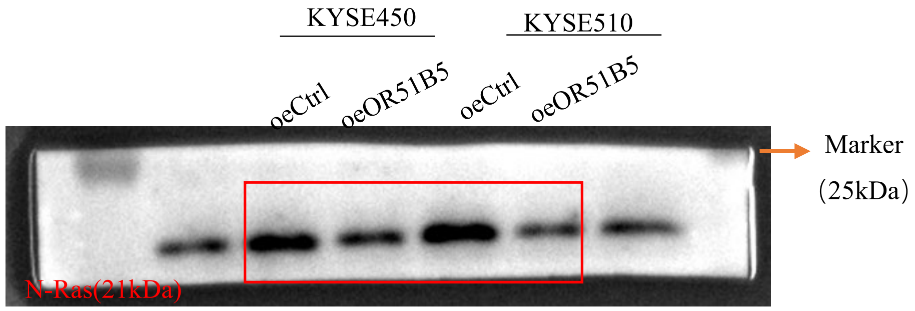

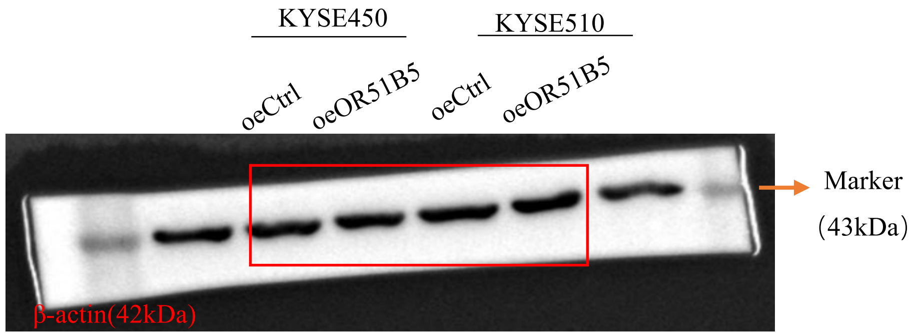

Original western blots Figure 7A

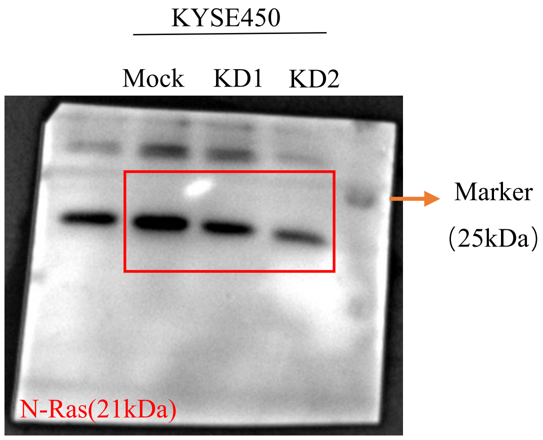

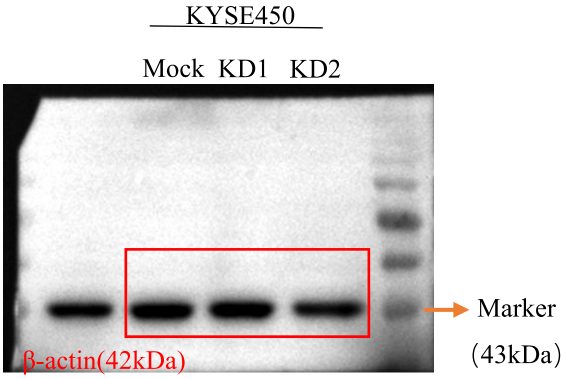

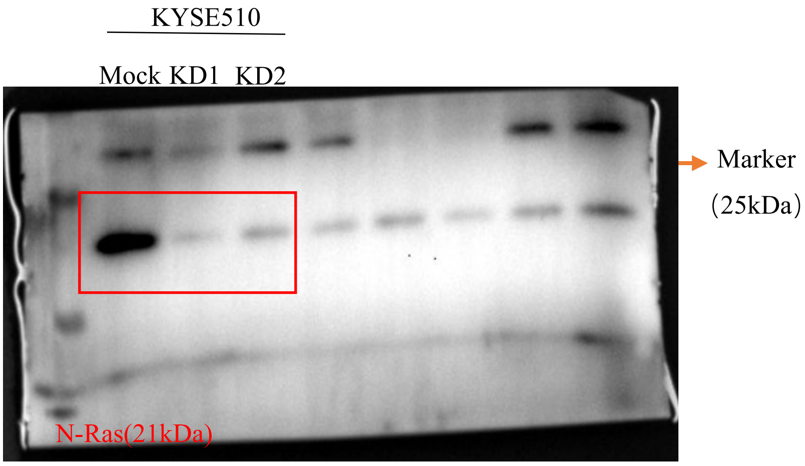

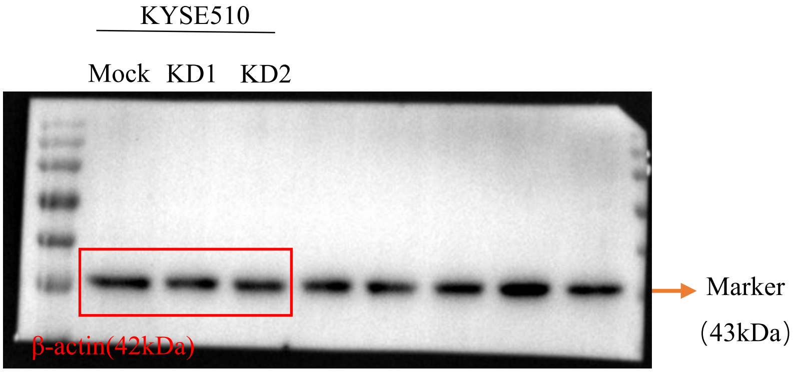

Original western blots Figure S1B

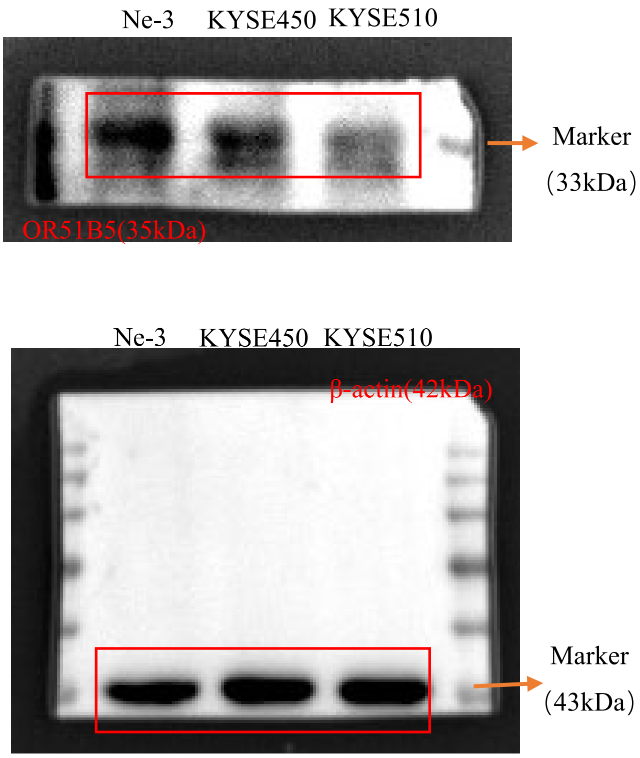

Original western blots Figure S1B

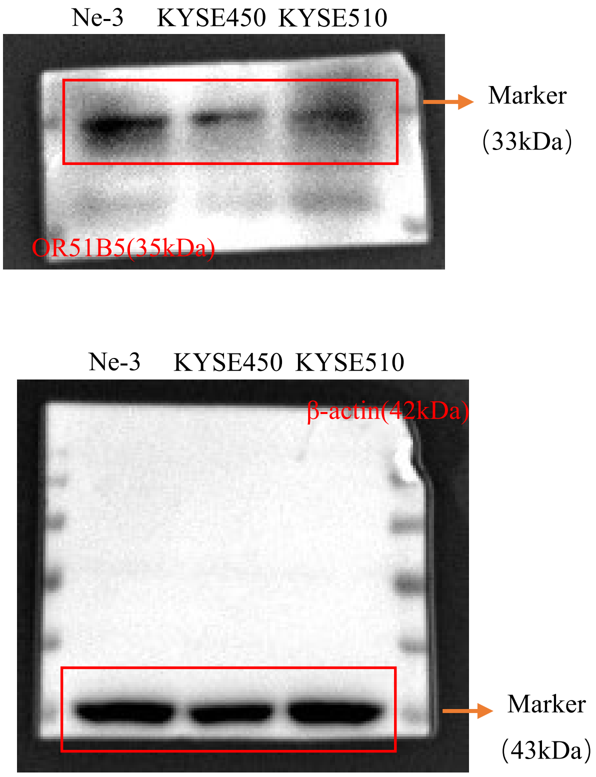

Original western blots Figure S2A

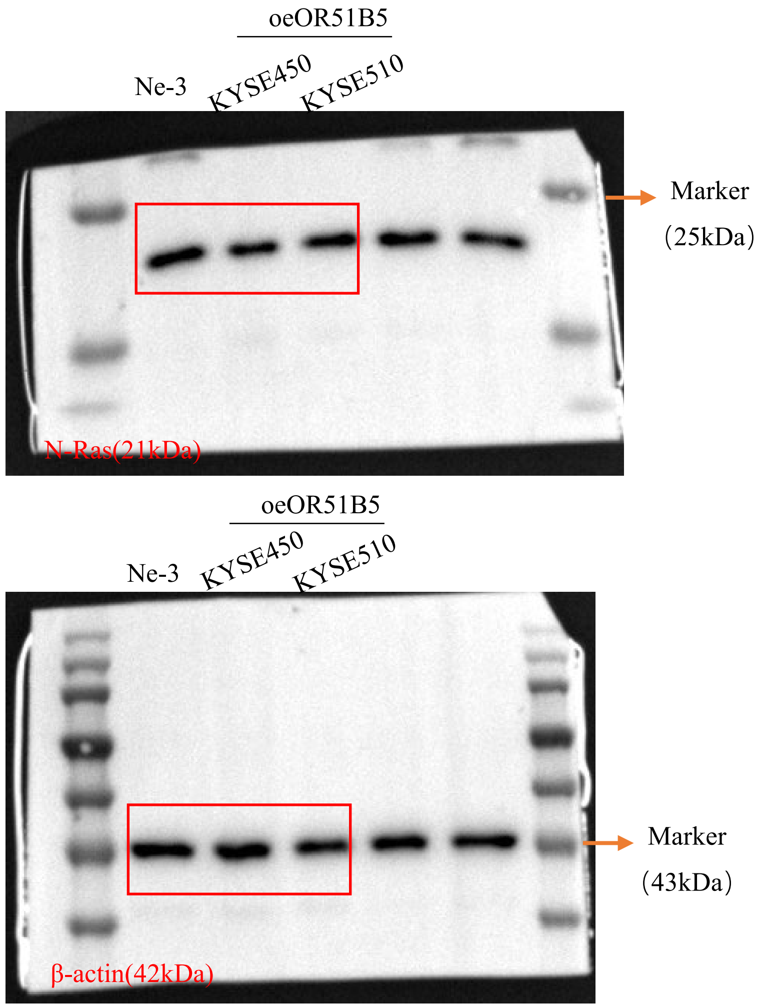

Original western blots Figure S2B

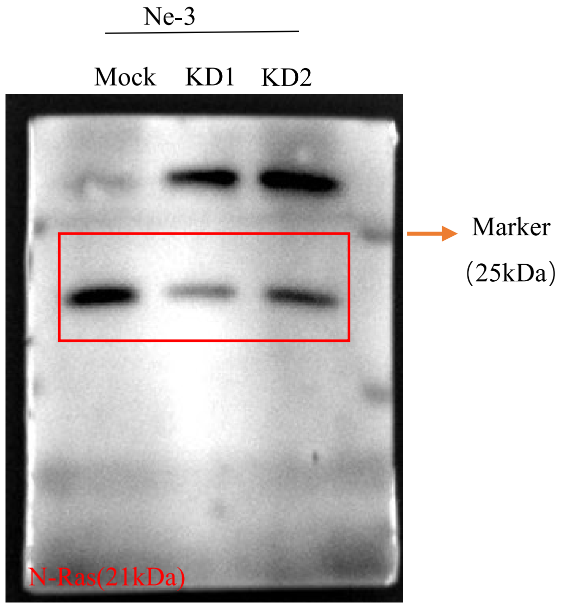

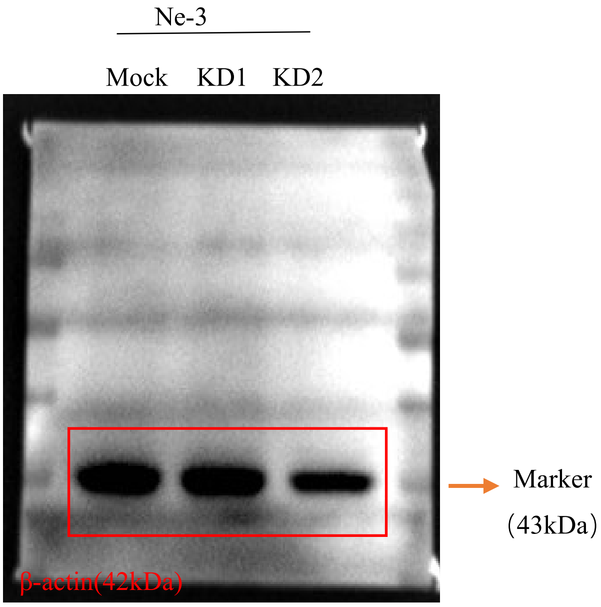
Ne-3
Mock
KD1
KD2
β-actin(42kDa)
Marker
(43kDa)
